# Comparative Transcriptome Analysis Demonstrates the Positive Effect of the Cyclic AMP Receptor Protein Crp on Daptomycin Biosynthesis in *Streptomyces roseosporus*

**DOI:** 10.3389/fbioe.2021.618029

**Published:** 2021-06-04

**Authors:** Jiequn Wu, Danqing Chen, Jinrong Wu, Xiaohe Chu, Yongmei Yang, Lina Fang, Wei Zhang

**Affiliations:** ^1^Collaborative Innovation Center of Yangtze River Delta Region Green Pharmaceuticals, College of Pharmaceutical Sciences, Zhejiang University of Technology, Hangzhou, China; ^2^Hangzhou Zhongmei Huadong Pharmaceutical Co., Ltd., Hangzhou, China

**Keywords:** daptomycin, cyclic lipopeptide antibiotic, *Streptomyces roseosporus*, NRPS, Crp, transcriptional regulator, transcriptome analyses

## Abstract

Daptomycin, which is produced by *Streptomyces roseosporus*, has been characterized as a novel cyclic lipopeptide antibiotic that is effective against Gram-positive bacteria. The biosynthesis of daptomycin is regulated by various factors. In the present study, we demonstrated that the cyclic AMP receptor protein (Crp) plays an important role in producing daptomycin in the *S. roseosporus* industrial strain. We found that daptomycin production from the *crp* deletion strain decreased drastically, whereas production from the *crp* overexpression strain increased by 22.1%. Transcriptome and qPCR analyses showed that some genes related to the daptomycin biosynthetic gene cluster (*dpt*) and the pleiotropic regulator (*adpA*) were significantly upregulated. RNA-seq also shows Crp to be a multifunctional regulator that modulates primary metabolism and enhances precursor flux to secondary metabolite biosynthesis. These results provide guidance for the development and improvement of potential natural products.

## Introduction

Cyclic lipopeptides (CLPs) are antibiotics widely used for the treatment of infections by Gram-positive organisms (Gotze et al., 2017), such as rapidly emerging antibiotic-resistant bacteria. Daptomycin is a CLP that is produced by *Streptomyces roseosporus* through a non-ribosomal peptide synthetase (NRPS) pathway. This CLP is classified as a last-resort antibiotic, along with vancomycin and linezolid (Gray and Wenzel, 2020), which was first reported by Eli Lilly and Company (Kirkpatrick et al., 2003) and was approved by the FDA in 2003 to treat skin infections and bacteremia caused by Gram-positive bacteria, especially methicillin-resistant *Staphylococcus aureus* (MRSA) (Nguyen et al., 2006). The biosynthetic gene cluster of daptomycin (*dpt*) has been identified and includes *dptA*, *dptBC*, *dptD*, *dptE*, *dptF*, *dptG*, *dptH*, *dptI*, *dptJ*, *dptP*, *dptM*, and *dptN* (Miao et al., 2005). The daptomycin biosynthesis pathway is initiated by the activation of decanoic acid by DptE (an acyl-CoA ligase). The acid is then transferred onto an acyl carrier protein (encoded by *dptF*) and coupled with the N-terminus of the tyrosinase related protein (Trp1) as a fatty acid side-chain (Wittmann et al., 2008). Trp1, and 12 amino acids, are then linearly assembled to form daptomycin *via* DptA, DptBC, and DptD, the three subunits of the NRPS pathway for amino acid condensation (Baltz, 2010). The non-proteinogenic amino acid precursors for the synthesis of daptomycin, L-3-methylglutamic acid, and Kyn (kynurenine), are formed by the genes *dptG*, *dptI*, and *dptJ* (Baltz et al., 2005; Wang et al., 2014), and it has been reported that *dptP*, *dptM*, and *dptN* may be involved in daptomycin resistance and/or export (Baltz, 2010).

Many regulatory factors that affect natural products have recently been reported, due to the discovery of biosynthetic gene clusters (Liu et al., 2018). These factors can be triggered by carbon sources, such as acyl-CoA; nitrogen sources, such as amino acids; and phosphate, among others. The cyclic AMP receptor protein (Crp) has been characterized as a typical global factor regulating sugar metabolism in *Escherichia coli*. Crp recognizes cyclic AMP (cAMP, a second messenger), and the Crp-cAMP complex formed under glucose starvation conditions. The transcriptional regulatory mechanism of the Crp-cAMP complex in *E. coli* has been thoroughly elucidated (Gosset et al., 2004). In both *E. coli* and *Streptomyces*, the homologous sequence for Crp has been identified (Angell et al., 1994; Derouaux et al., 2004a; Lin et al., 2020). The protein structure of Crp (PDB: 1HW5) has also been clearly identified in *E. coli*-derived bacteria (Won et al., 2009). The structure of Crp consists of two domains: a large N-terminal cAMP-accepting domain and a small C-terminal DNA-binding domain with a conserved helix-turn-helix motif (Fic et al., 2009). However, the mechanism of glucose repression in *Streptomyces coelicolor* is completely different from that in *E. coli* (Angell et al., 1994). Crp deficiency retards growth, which is consistent with the phenotype of the adenylate cyclase (*cya*) mutant that cannot synthesize cAMP (Derouaux et al., 2004b). In addition to morphological development, recent studies have demonstrated that Crp regulates the synthesis of a range of secondary metabolites, such as actinomycin (Act), undecylprodigiosin (Red), and calcium-dependent antibiotic (CDA) in *S. coelicolor* (Gao et al., 2012), and monensin in *Streptomyces cinnamonensis*, by enhancing acyl-CoA precursors and upregulating the transcription of biosynthetic gene clusters (Lin et al., 2020).

In this study, we aimed to identify Crp in the *S. roseosporus* industrial strain, SR1101, for the first time, and investigated the role of this receptor protein in primary metabolism and secondary metabolism. We hypothesized that there would be a different regulatory mechanism of Crp in *S. roseosporus* and that this protein may affect the yield of daptomycin in *S. roseosporus* as in other *Streptomyces* strains.

## Materials and Methods

### Media and Culture Conditions

*Escherichia coli* strains were cultured in Luria–Bertani medium (1.0% tryptone, 0.5% yeast extract, 1.0% NaCl) at 37.0°C and at 220 rpm. The original industrial strain of *S. roseosporus*, SR1101, was provided by Hangzhou Zhongmei Huadong Pharmaceutical Co., Ltd. (Hangzhou, China). *Streptomyces* was grown on R5 medium at 30.0°C for sporulation {1.0 L: 10.3% sucrose, 0.25‰ K_2_SO_4_, 1.0% MgCl_2_⋅6H_2_O, 1.0% glucose, 0.1‰ casein hydrolysate, 0.5% yeast extract, 57.3 mL of TES buffer, 10.0 mL of 0.5% KH_2_PO_4_, 4.0 mL of 5.0 M CaCl_2_, 20.0 mL of 20.0% L-proline, 7.0 mL of 1.0 N NaOH, 2.0% agar, 2.0 mL trace element solution [40.0 mg⋅L^–1^ ZnCl_2_, 200.0 mg⋅L^–1^ FeCl_2_⋅6H_2_O, 10.0 mg⋅L^–1^ CuCl_2_⋅2H_2_O, 10.0 mg⋅L^–1^ MnCl_2_⋅2H_2_O, 10.0 mg⋅L^–1^ Na_2_B_4_O_7_⋅10H_2_O, 10.0 mg⋅L^–1^ (NH_4_)_6_Mo_7_O_24_⋅4H_2_O]}. MS medium was used for conjugation (2.0% mannitol, 2.0% soya flour, 2.0% agar, 10.0 mM MgCl_2_) (Kieser et al., 2000). Shake flask fermentation was used to measure the daptomycin yield, in which TSB was used as seed medium (3.0% Oxoid tryptone soya broth power) and YEME as production medium (0.15% yeast extract, 0.5% tryptone, 0.3% malt extract, 1.0% glucose, and, 25.0% sucrose). *S. roseosporus* were precultured in TSB medium for 30 h at 30.0 °C and at 250 rpm. Next, 1.0 mL of the seed culture was introduced into 35.0 mL of the YEME fermentation medium for culturing for 4 days at 30.0°C and at 250 rpm. A feeding medium (Mao et al., 2017) containing decanoic acid and methyl oleate (1:1, v/v) with 0.2 g/L final concentration of decanoic acid was added at 48 h. The strains and plasmids used in this study are summarized in Supplementary Table 1.

### Construction of Plasmids and Mutant Strains

To construct *crp* deletion strain, in-frame gene deletion was performed using homologous recombination strategy. Two homologous fragments *crpL* and *crpR* flanking *crp* were amplified using the primer pairs CrpL-A/CrpL-S and CrpR-A/CrpR-S, and were digested with *Hind*III/*Xba*I and *Hind*III/*EcoR*I, respectively. The products were subsequently ligated with pOJ260 to construct the knockout plasmid pOJ260::*crpLR*. The *crp* knockout strain SR1160 was constructed by homologous recombination (Supplementary Figure 1A), in which the 1,065-bp *crp* gene region was completely deleted. This genotype was verified by PCR (Supplementary Figure 1B) with the primer pair CrpY-A/CrpY-S (Supplementary Table 2), where the wild type produced a 4,462-bp band, and the knockout showed a truncated 3,397-bp band.

The *crp* gene was amplified from *S*. *roseosporus* genomic DNA using the primer pair CrpA/CrpS, digested with *BamH*I and *Xba*I, and the resulting 858-bp *crp* fragment was cloned into an overexpression plasmid pZL2004. This integrative plasmid (pZL2004) is a pSET152 derivative carrying a 0.5-kb fragment containing the constitutive promoter *ermEp*^∗^ (Wu et al., 2011), and was used to create the plasmid pSET152::*ermepcrp*. The overexpression strain SR1130 was constructed *via*ΦC31 site-specific recombination (Supplementary Figures 1C,D) for the integration of an additional copy of the *crp* gene controlled under the strong promoter *ermEp*^∗^. The *crp* overexpression strain, SR1130, and knockout strain, SR1160, were obtained for subsequent fermentation and transcriptional analysis. The primers are listed in Supplementary Table 2.

### High Performance Liquid Chromatography Analysis of Daptomycin

After fermentation, the culture was centrifuged at 4,000 rpm for 10 min, and the supernatant was filtered through a 0.22-μm Millipore membrane. A high-performance liquid chromatography analysis of daptomycin with a 20.0-μL injection volume was performed using a C18 reverse-phase column (Welch Ultimate AQ-C18; 4.6 mm × 250 mm, 5 μm) with isocratic elution with solution A (0.05 M Na_2_HPO_4_, pH 3.15) and solution B (100% acetonitrile) at a ratio of 65:35, at a 214 nm UV detection wavelength and flow rate of 1.0 mL/min. Pure daptomycin was utilized as a standard (purity ≥ 98%, Aladdin).

### Biomass Analysis

To determine the biomass of WT and mutant strains, 10 mL fermentation broth was filtered with a Brinell funnel and washed twice with sterile water. The mycelium was then put into an oven at 60.0°C for drying and determine the dry cell weight.

### RNA Analysis

Total RNA was extracted from mycelia of strains SR1101 and SR1130 fermented in YEME medium for 3 days using a HiPure Bacterial RNA Kit (MAGEN) according to the manufacturer’s protocol. The fermentation broth was centrifuged and the mycelium was collected, mixed with Trizol at room temperature for 5 min, and then stored in liquid nitrogen for subsequent RNA extraction. The concentration of RNA was measured by UV detection at 260 nm, with ddH_2_O as the blank control. The purity of the RNA samples based on the ratio of OD_260/280_ ranged from to 1.8–2.2. The RNA samples were then processed and sequenced by LC-Bio Technology Co. (Hangzhou, China). The transcription levels of the genes were analyzed, and further information was obtained from the LC-Bio cloud platform^1^, such as the visual scatter plot and the volcano map of differentially expressed genes (DEGs). The threshold to identify DEGs was set at log2(FC) ≥ 1 (FDR ≤ 0.05). Goatools software was used to analyze the gene ontology (GO) enrichment of DEGs *via* a Fisher’s exact test for gene function prediction and classification. The p-value was corrected using multiple test methods to control the calculated false positive rate. When p-value ≤ 0.05, the GO term was considered to have a significant enrichment. KOBAS software was used to test the enrichment of DEGs in the Kyoto Encyclopedia of Genes and Genomes (KEGG) pathways for secondary metabolic pathway annotation, and the same calculation principle was used as that for GO enrichment analysis.

### Quantitative Real-Time PCR

The same amount of 800 ng RNA template was reverse-transcribed into cDNA using the RevertAid H Minus First Strand cDNA Synthesis Kit (Fermentas, Shanghai, China). The synthesized cDNA was diluted tenfold for subsequent qPCR analysis with 2 × ChamQ SYBR qPCR Master Mix, and the qPCR program setup was shown as follows: 95°C for 3 min; 40 cycles of 95°C for 10 s, and 60°C for 30 s; 95°C for 15 s, 60°C for 60 s and 95°C for 15 s. The fold change of expression level of SR1130 compared to SR1101 was calculated according to the cycle threshold (Ct value). The primers used in qCPCR are listed in Supplementary Table 2.

### Phylogenetic Analysis of Crp

To analyze the characteristics of Crp in *S. roseosporus*, we compared the homology of *crp* from 59 different sources, including *Streptomyces*, *E. coli*, and mycobacteria, by means of the Construct/Test Maximum Parsimony Tree(s) analysis in MEGA-X software.

## Results

### Phylogenetic Analysis of Crp

**FIGURE 1:**
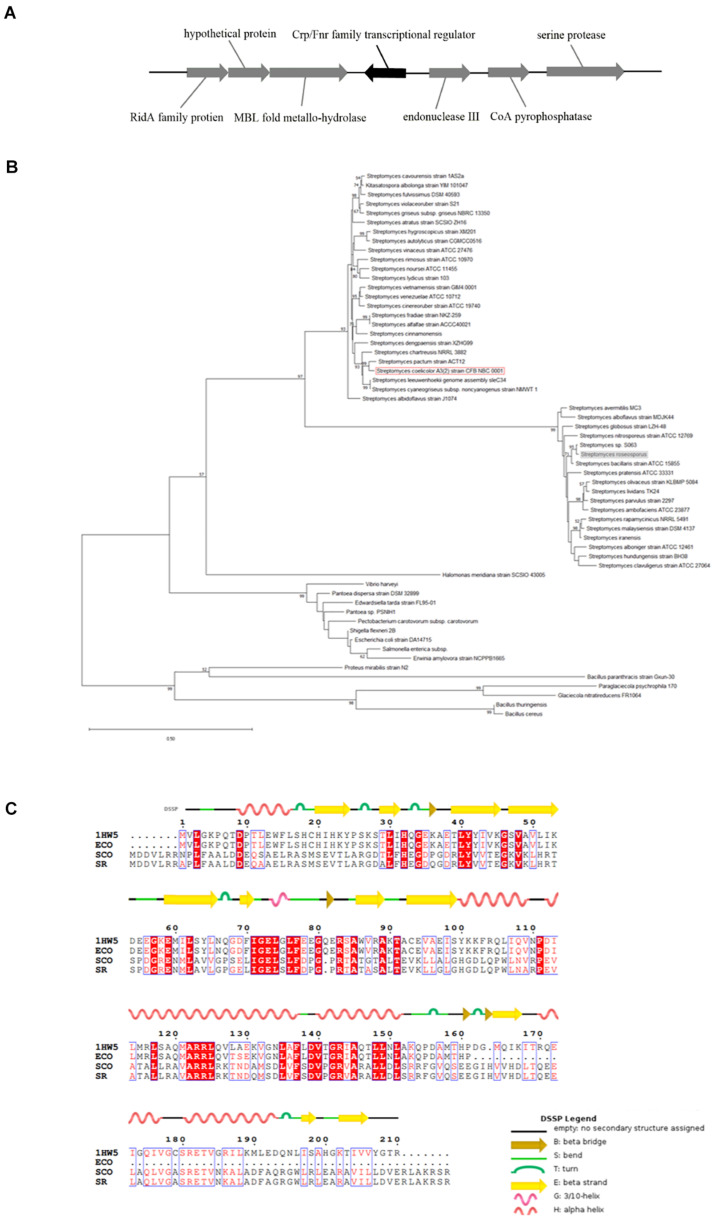
Crp has a highly conserved sequence and structure. **(A)** The locus of *crp* in *S. roseosporus*. **(B)** Phylogenetic analysis of 59 *crp* genes from different strains of bacteria (mycobacteria, *E. coli*, and *Streptomyces*). The *crp* of *S. coelicolor A3(2)* is shown in the red box, and that of *S. roseosporus* is in the gray box. **(C)** ClustalW analysis of Crp from *S. roseosporus*, *S. coelicolor A3(2)*, and *E. coli* with 1HW5. ECO: *E. coli*, SCO: *S. coelicolor A3(2)*, SR: *S. roseosporus*, 1HW5: Crp protein with known structure. The scale bar indicates the branch length.

The homologous *crp* gene (SSIG_RS14575) was found in *S. roseosporus* (Figure 1A) *via* a protein blast that used the Crp protein sequence of *S. coelicolor*. This sequence is a monocistron that contains 675 nucleotides (nt) and encodes a protein of 224 amino acids. The *crp* gene of *S. roseosporus* belonged to the same evolutionary branch as that of *S. coelicolor* (Figure 1B), and 89.3% (nucleotide) and 94.2% (amino acid) sequence identities were observed between *S. roseosporus* and *S. coelicolor*. In addition, the amino acid sequences (Figure 1C) show that the advanced structure was highly conserved between *S. coelicolor*, *S. roseosporus*, and *E. coli*. These findings show that Crp is a ubiquitous protein, and the presence of homologs in *S. coelicolor* and *S. cinnamonensis* suggest a conserved role in the genus.

### Role of Crp in the Daptomycin Industrial Production Strain

**FIGURE 2:**
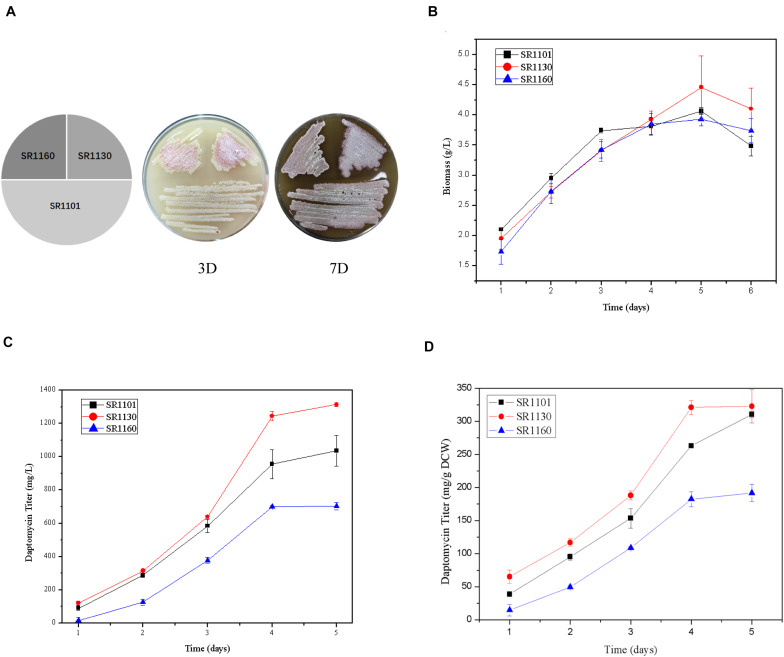
Crp has a positive effect on daptomycin yield but no effect on morphology. **(A)** Phenotypes of SR1101, SR1130, and SR1160 on R5 medium on days 3 and 7. The time curves of the **(B)** Biomass (dry cell weight) and **(C)** Daptomycin titer (mg/L) of the parental strain SR1101, the *crp* overexpression strain SR1130, and the *crp* knockout strain SR1160. **(D)** Production comparison of daptomycin (mg/g DCW) among the strains SR1101, SR1130, and SR1160. Data represent the means ± standard deviations from three independent experiments.

The strains SR1101, SR1130, and SR1160, streaked on R5 medium, show that sporulation and pigment production of the mutant strains on days 3 and 7 were similar to those of the parental strain SR1101 (Figure 2A). This indicates that the mutation had no effect on morphological differentiation in *S. roseosporus*.

Fermentation was carried out to detect the biomass and daptomycin production at one-day intervals. There were no significant differences among the three strains in dry cell weight (Figure 2B). These biomass curves implied that Crp did not affect the growth of *S. roseosporus*.

Furthermore, the yield of the overexpressed strain SR1130 in the first 3 days was slightly higher than that of strain SR1101, but there was a marked increase of 22.1% on day 4 (Figures 2C,D). In addition, the daptomycin yield of the *crp* deletion strain, SR1160, decreased by approximately 30% compared to that of SR1101 during fermentation (Figures 2C,D). The results indicate that Crp promoted the biosynthesis of daptomycin during the late stage of fermentation, but it was not caused by mycelium growth.

### The Influence of Crp on the Transcriptional Level of dpt Cluster

**FIGURE 3:**
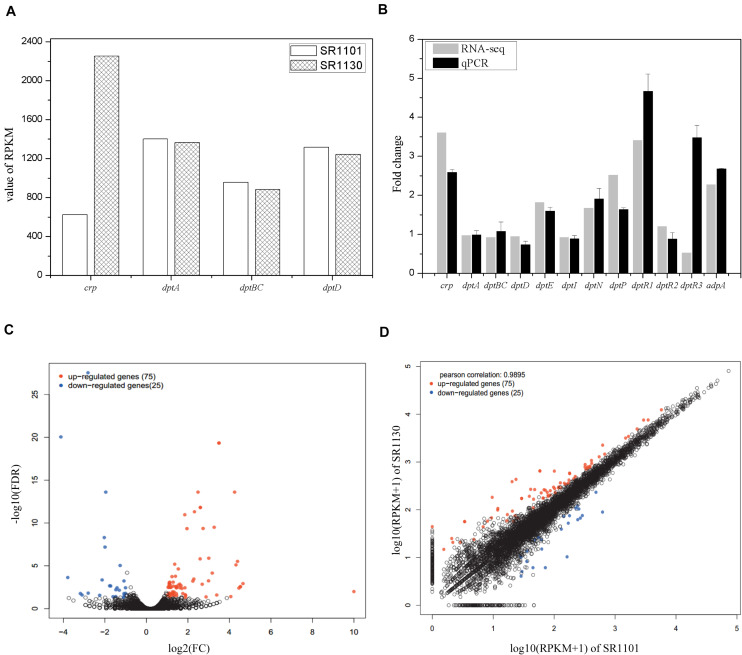
Transcription comparisons between the parental strain SR1101 and overexpression strain SR1130. **(A)** The transcription level of *crp* and NRPSs in SR1101 and SR1130 (RPKM value). **(B)** The relative expression level of genes measured by RNA-seq and qPCR analysis. The Fold change of each gene was normalized with that of SR1101. The sigma factor gene *hrdB* was used as an internal control in qPCR analysis. Data represent the means ± standard deviations from three independent experiments. **(C)** Volcano map of DEGs: The horizontal axis represents the fold change in gene expression between two samples, whereas the vertical axis represents the statistical significance of the gene expression difference. **(D)** Scatter plot: The horizontal and vertical coordinates represent the gene or transcript expression in the two samples, respectively. Red dots indicate significantly upregulated genes, blue dots indicate significantly downregulated genes, and black dots indicate non-significantly different genes.

To investigate the regulatory mechanisms that govern the effect of Crp on daptomycin biosynthesis, RNA-seq of SR1101 and SR1130 was performed. Based on the time curve of the daptomycin titer (Figures 2C,D), we hypothesized that Crp interfered with the overall transcription rates on approximately day three. The fermentation broths from the third to the fourth day were therefore used to extract RNA samples for RNA-seq and qPCR analysis. The transcriptional level of the *crp* gene (SSIG_RS14575) was compared to SR1101. This revealed that the overexpression strain, SR1130, increased *crp* transcription by 3.6-fold in reads per kilobase of transcript per million reads mapped (RPKM) (Mortazavi et al., 2008), and 2.5-fold in qPCR analysis, indicating that we had successfully constructed the *crp* overexpression strain (Figures 3A,B).

The expression level of genes involved in the synthesis of daptomycin were compared. We found that *crp* overexpression did not lead to an increase in the transcription of the NRPS-encoding genes in *dpt* (*dptA*, *dptBC*, or *dptD*) (Figures 3A,B). Thus, we inferred that Crp does not directly affect NRPS pathways, but may have an alternative function. To identify the underlying cause, we analyzed the transcription of the whole *dpt* cluster and defined the transcription level of SR1101 as 1 (Supplementary Figure 2). qPCR was then performed to verify the relative expression of some genes (Figure 3B). The transcriptional level of *dptE* and *dptF* increased by 1.8 folds. These results were consistent with qPCR data. The *dptMN* genes (encoding the ABC transporter, SSIG_RS30380, and SSIG_RS30385) and *dptP* gene (encoding the DedA-family protein, SSIG_RS30370) involved in daptomycin resistance and/or export (Baltz, 2010) were found to be upregulated 1.7-fold to 2.5-fold in SR1130 when compared with those of SR1101 (Figure 3).

To determine whether Crp affected daptomycin yield by regulating the regulatory genes, DptR1, DptR2, and DptR3, we compared the transcriptional levels of *dptR1*, *dptR2*, and *dptR3* in the *crp* overexpression strain, SR1130, and original strain, SR1101. As shown in Figure 3B, the transcription levels of *dptR1* were increased in both RNA-seq and qPCR results. There was little difference in gene expression levels of *dptR2* between SR1101 and SR1130. However, qPCR analysis showed that the transcription level of *dptR3* increased by 3.5-fold in SR1130 compared with that in SR1101, which contradicted the results of RNA-seq. These findings indicated that Crp may strongly stimulate the expression of *dptR1* and *dptR3*. In addition, AdpA (SSIG_RS22080), a positive regulator of daptomycin biosynthesis (Mao et al., 2015), was significantly upregulated in the *crp* overexpression strain (Figure 3B), which could be a reason for the production enhancement.

### Summary of RNA-Seq Data and Functional Analysis of DEGs

**FIGURE 4:**
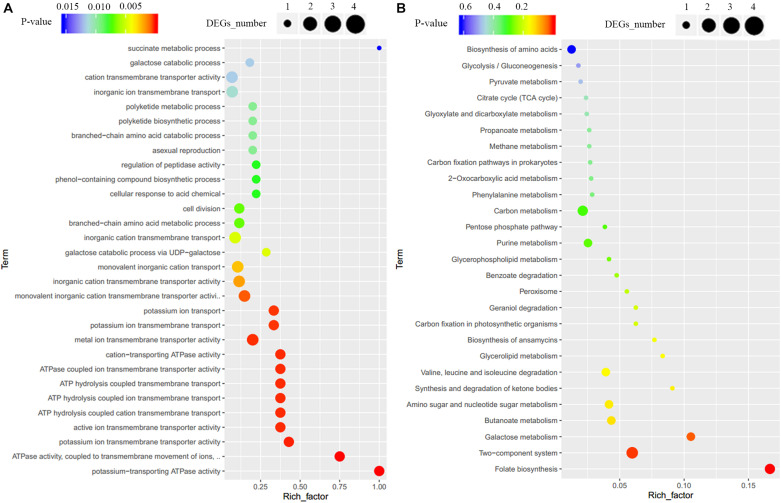
GO enrichment and KEGG enrichment of DEGs. **(A)** GO enrichment. **(B)** KEGG enrichment. The horizontal axis represents the enrichment factor, that is, the ratio of the number of DEGs enriched to the number of background genes obtained by sequencing. The vertical axis represents the function of the GO term or KEGG pathway. The larger the circle is, the greater the number of DEGs enriched with this function. The spectrum from blue to red represents the uncorrected P-values.

To clarify the effect of Crp on other biosynthetic pathways, DEGs were characterized online (see text footnote 1). DEGs were between the original strain, SR1101, and *crp* overexpression strain, SR1130, as displayed by the volcano map (Figure 3C) and the visual scatter plot (Figure 3D). We found that 75 genes were upregulated, and 25 genes were downregulated in SR1130 compared with those in strain SR1101 (Supplementary Table 3). The functions of these 100 DEGs were then investigated using GO enrichment (Figure 4A) and KEGG enrichment (Figure 4B). The genes were divided into three general categories in the GO database: biological process (BP), cellular component (CC), and molecular function (MF). As shown in Figure 4A, the gene sets involved in the ATP-hydrolysis-coupled transmembrane transport (GO:0090662), branched-chain amino acid metabolic process (GO:0009081), polyketide biosynthetic process (GO:0030639), galactose catabolic process (GO:0019388), and cell division (GO:0051301) in the BP category were significantly enriched. GO terms such as serine-type endopeptidase complex (GO:1905370) and CD40 receptor complex (GO:0035631) were enriched in the CC category. In the MF category, gene sets in the potassium-transporting ATPase activity (GO:0008556), ATP adenylyltransferase activity (GO:0003877), purine ribonucleotide binding (GO:0032555), glucose transmembrane transporter activity (GO:0005355), and acyl-CoA ligase activity (GO:0003996) were significantly enriched. These results indicate that Crp exerts a fundamental regulatory function in *S*. *roseosporus*.

When the 100 DEGs were mapped to KEGG pathways, primary metabolism including folate biosynthesis, the two-component system, and galactose metabolism, were found to be effectively regulated by Crp (Figure 4B). The DEG galE (SSIG_RS02835, Supplementary Table 3) enriched in galactose metabolism and nucleotide sugar metabolism was upregulated 3.7-fold, and is involved in the formation of UDP-glucose to synthesize 3-amino-5-hydroxybenzoic acid (AHBA), an aromatic starter unit of the biosynthesis of rifamycin (Floss and Yu, 2005).

The genes SSIG_RS11485 (encoding potassium-transporting ATPase subunit KdpA), SSIG_RS11480 (encoding potassium-transporting ATPase subunit B), SSIG_RS11475 (encoding potassium-transporting ATPase subunit C), and SSIG_RS06275 (encoding sensor histidine kinase) were significantly upregulated (Supplementary Table 3) and enriched in the two-component system category (KO: 02020). For carbon metabolism, 29 DEGs, including the genes encoding the acyl-CoA synthetase, methylmalonyl-CoA epimerase, succinate-CoA ligase, acetyl-CoA C-acetyltransferase, and ADP-forming succinate-CoA ligase, were upregulated. These could provide basic units for the synthesis of secondary metabolites in *Streptomyces*.

## Discussion

**FIGURE 5:**
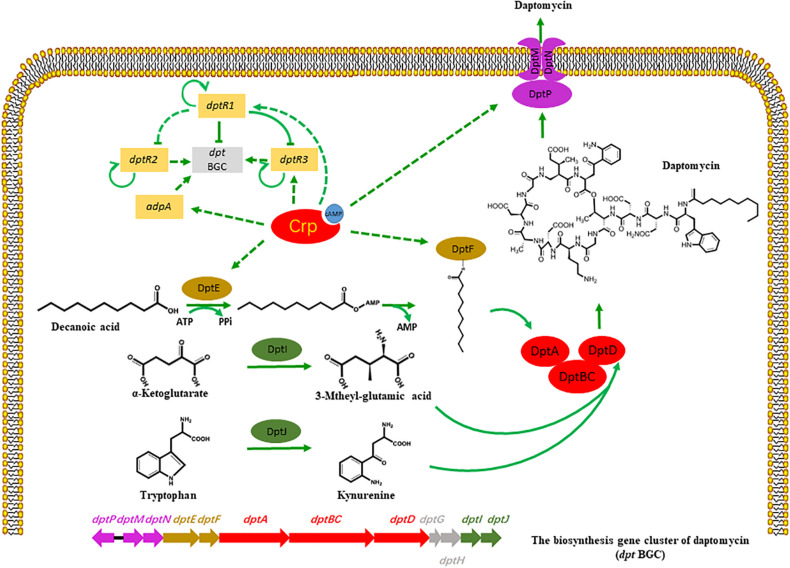
Potential model of regulatory role of Crp on daptomycin production. Bars: repression. Solid arrows: activation. Solid lines: direct regulation. Dashed lines: indirect regulation or unknown route.

Cyclic AMP receptor protein is broadly distributed in a variety of bacteria and regulates multiple biological activities, such as glucose starvation, cell differentiation, and primary metabolism (Shimada et al., 2011). As Crp is a highly conserved regulatory protein in *Streptomyces*, we aimed to detect the homologous Crp protein in the industrial strain *S. roseosporus*, SR1101, *via* evolutionary analysis and protein alignment. A Crp deletion, and a Crp overexpression, decreased, and increased, the production of daptomycin, respectively, demonstrating that Crp plays a significant role in the synthesis of daptomycin. Analysis of the transcriptome data of SR1101 and SR1130 shows that Crp acted in two ways (Figure 5):

(I) Crp regulated the expressions of some *dpt* genes during daptomycin production. The increased transcription levels of *dptE* and *dptF* in the *crp* overexpression strain, which are responsible for the activation and acylation of fatty acid precursors, enhanced the utilization of the decanoic acid precursor and caused the yield to increase. This phenomenon is supported by a previous study, where the overexpression of *dptEF* resulted in a 3.9-fold increase in daptomycin production (Lee et al., 2016). The transcription levels of *dptH*, *dptI*, and *dptJ*, which are primarily involved in amino acid modification or the formation of L-3-methylglutamic acid and Kyn (Baltz, 2010; Liao et al., 2013), were similar between SR1101 and SR1130, suggesting that the supply of non-proteinogenic amino acid precursors had not been enhanced. Another study found that the introduction of an additional copy of *dptI* enhanced the production of daptomycin by 110 % to enhance the supply of Kyn (Liao et al., 2013). Crp also increased the expression of *dptP*, *dptM*, and *dptN*, which appeared to be favorable for daptomycin transport and/or resistance. According to the transcriptional data for *dpt*, the improvement in daptomycin production might result from the increased amount of decanoic acid, rather than the amino acids. In addition, enhancing the efflux and/or resistance of daptomycin may promote synthesis.

(II) Crp had a temporary indirect effect on daptomycin yield by upregulating DptR1, DptR3, and AdpA. The upregulated DptR1 in strain SR1130 may have led to a decline in the daptomycin yield. However, the positive regulator DptR3 and AdpA may have counteracted this negative effect, which would explain why the yield did not significant increase on day 3. When the A-factor receptor protein (ArpA) couples with A-factor, it acts directly to promote *adpA*, and stimulates a range of genes involved in phenotypic growth and secondary metabolism (Ohnishi et al., 2014). These findings suggest that Crp stimulated daptomycin yield by regulating various transcriptional factors.

The global regulator Crp protein regulates not only the core genes of the secondary metabolite biosynthetic gene cluster, but also the cluster-situated regulatory gene (*monRI*) in *S*. *cinnamonensis* (Lin et al., 2020). In *S*. *roseosporus*, *dptR1*, *dptR2*, and *dptR3* are located adjacent to the *dpt* gene cluster, which leads to their description as potential cluster-situated regulators (Miao et al., 2005). A recent study showed that DptR1 decreases the yield of daptomycin, increases the transcriptional level of the core *dpt* genes on day 3, and decreases the transcriptional level of the core *dpt* genes on day 4. DptR1 also represses the transcription of DptR2 indirectly, and the transcription of DptR3 directly (Yu et al., 2020). DptR2, a DeoR-type autoregulator, is required for daptomycin production but not for the expression of NRPS genes (Wang et al., 2014). DptR3, belonging to the MarR family regulator, stimulates daptomycin production indirectly by altering the transcription of genes in *dpt* cluster (Zhang et al., 2015).

The interaction of multiple regulatory factors, as well as the repression/activation of the *dpt* cluster, might explain why the production of SR1130 was not much higher than that of the wild type in the first 3 days, but then significantly increased on day 4 of fermentation. The upregulated levels of AdpA in this study may have been one of the reasons for the increased yield of daptomycin, but no significant difference was found between the mutant strain and the original strain in terms of phenotype. This indicates that the mutation had no effect on morphological differentiation in *S. roseosporus*, which is not consistent with the sporulation deficiency observed in *S. coelicolor* (Derouaux et al., 2004b).

Comparative transcriptome analysis revealed that Crp, in addition to its influence on daptomycin biosynthesis, is involved in many types of primary metabolism, such as the two-component system and carbon metabolism, and would provide energy and extension units for the synthesis of other secondary metabolites (Figure 4 and Supplementary Table 3). A two-component system has been reported to be essential in *S. roseosporus* for complex regulation processes (Liao et al., 2012).

A series of methods are currently available to improve the yield of daptomycin in *S. roseosporus*. For example, the TetR-family transcriptional regulator, DepR1, directly and positively regulates daptomycin production by binding to the promoter of *dptE* (Yuan et al., 2016). The direct interaction of the ArsR-family regulator DepR2 with *dptEp* has also been confirmed, where the deletion of *depR2* increases the daptomycin yield by 150% (Mao et al., 2017). The role of the global regulator AtrA in antibiotic production in *S. roseosporus* has been reported (Mao et al., 2015). AtrA activates the transcription of *dptE* and increases the production of daptomycin under the controlled of AdpA. A member of the WhiB-family, WblA, has also been identified (Huang et al., 2017). A WblA knockout strain increases daptomycin yield by 51% but inhibits spore differentiation and growth. However, BldD has an opposite effect to WblA, as BldD acts as a repressor of the development of *S. roseosporus*, but an enhancer for daptomycin production, leading to ∼35% greater yields than those of the overexpression strain ObldD (Yan et al., 2020). Interestingly, a ribosomal protein, L3, with a G152V mutation, that is encoded by *rplC*, shows increased production of daptomycin in comparison to that of the wild type strain owing to enhanced gene transcription of the daptomycin biosynthetic genes (Li et al., 2013). These engineering methods combined with Crp overexpression found herein form a complex regulatory process that may enable the enhancement of the daptomycin yield.

This report describes the first beneficial attempt of Crp genetic modification in the industrial strain of *S. roseosporus* for daptomycin production. We show that this receptor protein plays a global role in primary and secondary metabolism. A deletion of the *crp* gene in *S. roseosporus* dramatically decreased the yield of daptomycin but did not cause a morphological change, whereas an engineered *crp* overexpression strain enhanced daptomycin biosynthesis and demonstrated a strong potential for industrial applications. Consequently, Crp was determined to be an overall regulator of primary metabolism that stimulates the production of secondary metabolites, such as polyketide and peptide compounds, in the daptomycin production industrial strain. The regulatory mechanism for Crp promoted daptomycin biosynthesis in *S. roseosporus* requires further investigation and could increase the understanding of metabolic engineering, which may be useful in industrial applications and development.

## Data Availability Statement

The datasets presented in this study can be found in online repositories. The names of the repository/repositories and accession number(s) can be found in the article/Supplementary Material.

## Author Contributions

JieW and JinW conceived the study. DC and JinW constructed the plasmids and strains and analyzed the data. DC wrote the manuscript. LF and YY performed the fermentation. JieW, XC, and WZ supervised and helped in the critical review and editing of the manuscript. All authors contributed to the article and approved the submitted version.

## Conflict of Interest

YY, LF, and WZ were employed by the company Hangzhou Zhongmei Huadong Pharmaceutical Co., Ltd. The remaining authors declare that the research was conducted in the absence of any commercial or financial relationships that could be construed as a potential conflict of interest.

## Footnotes

1 https://www.lc-bio.cn/analysis
