# Supplementary figures and images for: Comparative Transcriptome Analysis Demonstrates the Positive Effect of the Cyclic AMP Receptor Protein Crp on Daptomycin Biosynthesis in Streptomyces roseosporus
Source: Front Bioeng Biotechnol. 2021 Jun 4;9:618029. doi: 10.3389/fbioe.2021.618029 (PMC8212052; doi:10.3389/fbioe.2021.618029)

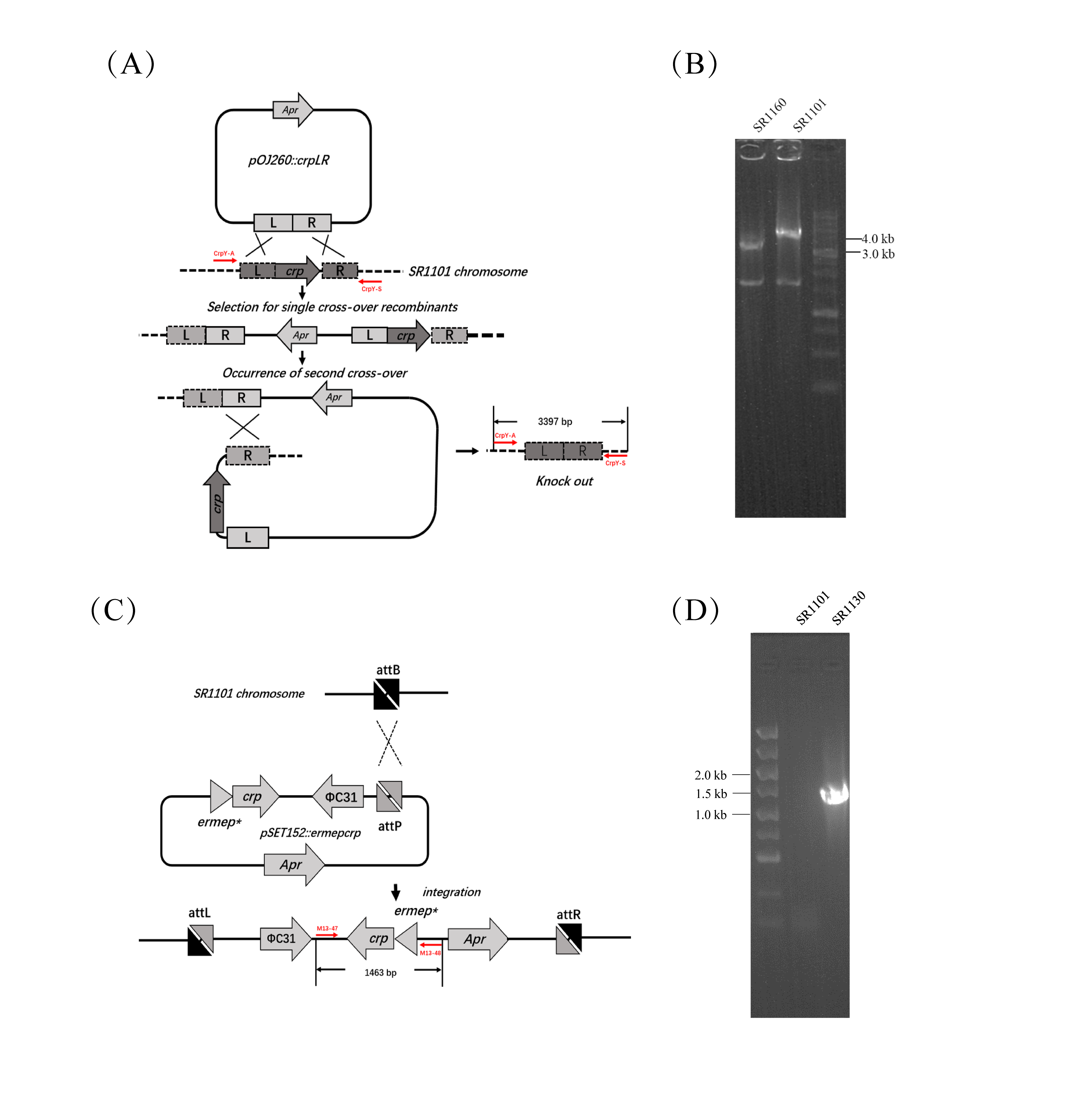

Supplement: Supplementary file 1 [file Image_1.TIF]
