# Supplementary material for: Comparative Transcriptome Analysis Demonstrates the Positive Effect of the Cyclic AMP Receptor Protein Crp on Daptomycin Biosynthesis in Streptomyces roseosporus
Source: Front Bioeng Biotechnol. 2021 Jun 4;9:618029. doi: 10.3389/fbioe.2021.618029 (PMC8212052; doi:10.3389/fbioe.2021.618029)
Supplement: Supplementary file 4 [file Data_Sheet_2.docx]

Supplementary Material

# Supplementary Data

# Supplementary Figures and Tables

## Supplementary Tables

**Supplementary Table S1.** Strains and plasmids used in this study

| **Strains or plasmids** | **Description** | **Source** |
| --- | --- | --- |
| Strains |  |  |
| SR1101 | *Streptomyces roseosporus* industrial stain for daptomycin production | Hangzhou Zhongmeihuadong Pharmaceutical Co., Ltd. (Hangzhou, China) |
| SR1130 | *crp* overexpression derivative of SR1101 with pSET152::*ermepcrp* integrative vector | This study |
| SR1160 | *crp* knockout derivative of SR1101 with pOJ260::*crpLR* vector | This study |
| *E.* *coli* DH5α | Host for gene cloning | Invitrogen |
| *E. coli* ET12567 (pUZ8002) | Donor strain for conjugation between *E.* *coli* and *Streptomyces* | ([Kieser et al., 2000](#_ENREF_2)) |
| Plasmids |  |  |
| pZL2004 | pSET152 derivative containing the constitutive promoter *ermEp**; Apr | ([Wu et al., 2011](#_ENREF_3)) |
| pOJ260 | *E. coli*-*Streptomyces* shuttle vector, Apr | ([Chen et al., 2016](#_ENREF_1)) |
| pSET152::*ermepcrp* | 858-bp *crp* fragment controlled by *ermEp** in pSET152 | This study |
| pOJ260::*crpLR* | 3038-bp fragment contained the homologous upstream and downstream sequence of *crp* in pOJ260 | This study |

**Supplementary Table S2.** Primer sequence in this study

| **Primer** | **Sequence** |
| --- | --- |
| CrpA/*Xba*Ⅰ | ATTTCTAGAAATACCTGACCGAGCAC |
| CrpS/*BamH*Ⅰ | ATTGGATCCATCGCACTGTTTTACCG |
| CrpL-A/*Hind*Ⅲ | ATTAAGCTTCGGTCAGGTATTCCAACT |
| CrpL-S/*Xba*Ⅰ | ATTTCTAGACCCGTTGGAGCACTG |
| CrpR-A/*EcoR*Ⅰ | ATTGAATTCGCGTCACGACGAAGC |
| CrpR-S/*Hind*Ⅲ | ATTAAGCTTGCGAATGGCTCCCTC |
| M13-47 | AgggTTTTCCCAgTCACg |
| M13-48 | gAgCggATAACAATTTCACAC |
| CrpY-A | GCCGTGAAACCCCAGACC |
| CrpY-S | CCGCCACGGTGATGCTGCTC |
| qPCR |  |
| dptR3-R | CCGGGTGGTCCGTATCGAGATC |
| dptR3-F | GCTGATCGGCGGTCAATGGAG |
| dptR2-R | ACCGCCCTGTTCGTCCACTC |
| dptR2-F | TGCCACCTCGTCGTCGTAGG |
| dptR1-R | GCCTGGTGCAGATGGATCAGATTG |
| dptR1-F | GTCACGAACGCCCTGGATCAC |
| crp-R | TGTTCCACGAGGGCGACCAG |
| crp-F | GAGCACGGCCAGCATGTTCTC |
| dptN-R | ACGAGGAACCCGATGCTGGAC |
| dptN-F | GTGGTCGCTGATCTGGGTGTTC |
| hrdB-R | ATCAGCGTCACACCCTCTTC |
| hrdB-F | AGTCCGAATCTGTGATGGCG |
| dptA-F | CGGCGTACATCATCCAGACC |
| dptA-R | GTCATGCTCAGTTCGGAGACG |
| dptBC-F | GAACAGACCACCCTCCTCG |
| dptBC-R | CTGTGGCCGATGGGGTAG |
| dptD-F | TTCCGGTACGAGCGGCTG |
| dptD-R | CGTCAGATCGAAGCGGCG |
| dptI-F | CTACCACGTGACCGTCAAGG |
| dptI-R | GTCGTCGAACTCGTTTCCCTC |
| dptP-F | CAATCCGCCGTACCAGGC |
| dptP-R | GGACACGCCCGCTGTTGG |
| adpA-F | GCTTAGCGTCATGAGCCAG |
| adpA-R | TGAACAGCAGCACGGCAA |

**Supplementary Table S3**. The DEGs fold changes of SR1130 compared with SR1101

## Supplementary Figures

**Supplementary Figure S1.** Construction of strains SR1160 and SR1130. (**A**) Knockout of the *crp* gene in the daptomycin industrial strain, SR1101, via homologous recombination. (**B**) The disruption mutant SR1160 was confirmed by PCR analysis using the primer CrpY-S and CrpY-A, which produced a 3397-bp band, whereas the wide type (SR1101) produced a 4462-bp band. The bands below 3.0 kb presented in both SR1101 and SR1160 were produced by a mismatch of the antisense primer (crpY-A). (**C**) The *crp* gene under the control of the constitutive promoter *ermEp** was introduced into SR1101 by ΦC31 site-specific recombination. (D) A 1463-bp band was obtained by PCR using the primer pair M13-47/M13-48 in pSET152::*ermepcrp*-integrated strain, SR1130, and SR1101 showed no bands. The sequencing results were stored in *Figshare* with DOI: https://doi.org/10.6084/m9.figshare.13487400.

**Supplementary Figure S2.** The relative expression of the whole *dpt* genes in SR1130 compared to SR1101 in RNA-seq. The RPKM value of SR1101 was defined as 1, as shown by the black dotted line.

# Reference styles

Chen, C., Hong, M., Chu, J., Huang, M., Ouyang, L., Tian, X., et al. (2016). Blocking the flow of propionate into TCA cycle through a mutB knockout leads to a significant increase of erythromycin production by an industrial strain of Saccharopolyspora erythraea. *Bioprocess and Biosystems Engineering* 40(2)**,** 201-209. doi: 10.1007/s00449-016-1687-5.

Kieser, T., Bibb, M.J., Buttner, M.J., Chater, K.F., and Hopwood, D.A. (2000). *Practical Streptomyces Genetics.*

Wu, J., Zhang, Q., Deng, W., Qian, J., Zhang, S., and Liu, W. (2011). Toward improvement of erythromycin A production in an industrial Saccharopolyspora erythraea strain via facilitation of genetic manipulation with an artificial attB site for specific recombination. *Appl Environ Microbiol* 77(21)**,** 7508-7516. doi: 10.1128/AEM.06034-11.
